# Severe paraneoplastic hypoglycemia in a patient with a gastrointestinal stromal tumor with an exon 9 mutation: a case report

**DOI:** 10.1186/1471-2407-7-13

**Published:** 2007-01-17

**Authors:** Guillermo A Escobar, William A Robinson, Trevor L Nydam, Drew C Heiple, Glen J Weiss, Linda Buckley, Rene Gonzalez, Martin D McCarter

**Affiliations:** 1 Department of Surgery, Division of GI, Tumor and Endocrine Surgery, University of Colorado Health Sciences Center. 4200 E. 9^th ^Ave, Denver, CO, 80262, USA; 2 Department of Medicine, Divisions of Medical Oncology and Endocrinology, University of Colorado Health Sciences Center. 4200 E. 9^th ^Ave, Denver, CO, 80262, USA

## Abstract

**Background:**

Non-islet cell tumor induced hypoglycemia (NICTH) is a very rare phenomenon, but even more so in gastrointestinal stromal tumors. It tends to present in large or metastatic tumors, and can appear at any time in the progression of the disease. We present herein a case of NICTH in a GIST tumor and report an exon 9 mutation associated to it.

**Case presentation:**

A thirty nine year-old man with a recurrent, metastatic gastrointestinal stromal tumor presented to the hospital with nausea, dizziness, loss of consciousness, and profound hypoglycemia (20 mg/dL). There was no evidence of factitious hypoglycemia. He was stabilized with a continuous glucose infusion and following selective vascular embolization, the patient underwent debulking of a multicentric 40 cm × 25 cm × 10 cm gastrointestinal stromal tumor. After resection, the patient became euglycemic and returned to his normal activities. Tumor analysis confirmed excessive production of insulin-like growth factor II m-RNA and the precursor protein, "big" insulin-like growth factor II. Mutational analysis also identified a rare, 6 bp tandem repeat insert (gcctat) at position 1530 in exon 9 of KIT.

**Conclusion:**

Optimal management of gastrointestinal stromal tumor-induced hypoglycemia requires a multidisciplinary approach, and surgical debulking is the treatment of choice to obtain immediate symptom relief. Imatinib or combinations of glucocorticoids and growth hormone are alternative palliative strategies for symptomatic hypoglycemia. In addition, mutations in exon 9 of the tyrosine kinase receptor KIT occur in 11–20% of GIST and are often associated with poor patient outcomes. The association of this KIT mutation with non-islet cell tumor induced hypoglycemia has yet to be established.

## Background

Gastrointestinal stromal tumors (GIST) are rare mesenchymal tumors most commonly found in the stomach or small intestine of adults. Their incidence ranges from 2.1 to 15 per million people, they are evenly distributed between genders, and 90% are CD-117 positive [1-3]. They usually grow asymptomatically until they are discovered due to appearance of signs and symptoms related to mass effects, bleeding or bowel perforation [4-6]. Other very rare clinical manifestations of GIST include hyperpigmentation or mastocytosis (both found in inherited forms of GIST) [7,8] and hypoglycemia [9-12].

Expression of CD-117 is the diagnostic marker for GIST and is the transmembrane tyrosine kinase receptor also known as KIT or c-KIT [13]. The ligand for the receptor is stem-cell factor, which is essential for embryonic development and proliferation of a variety of cell types including melanocytes, gonadal cells, and interstitial cells of Cajal (GI tract pacemaker cells) [14]. The latter are thought to be the physiological precursors to gastrointestinal stromal tumors and their proliferation may be due to unregulated activation of KIT [15].

To follow is a unique case associating GIST-induced hypoglycemia due to overproduction of "big" insulin-like growth factor II with an exon 9 mutation.

## Case presentation

A 39 year-old man with a multifocal abdominal recurrence of a small bowel gastrointestinal stromal tumor presented to the hospital following loss of consciousness preceded by weakness, dizziness and nausea. His serum glucose was 20 mg/dL (normal 80–110 mg/dL) and required a continuous infusion of high dose (10%) dextrose to maintain a serum glucose concentration above 70 mg/dL. Serologic workup demonstrated no evidence of factitious hypoglycemia and it discarded the diagnosis of islet cell-induced hypoglycemia. The insulin like growth factor (IGF) I and IGF binding protein 3 level were below the normal range, while insulin, pro-insulin, C-peptide and IGF II levels were all in the normal range (table 1).

**Figure 1:**
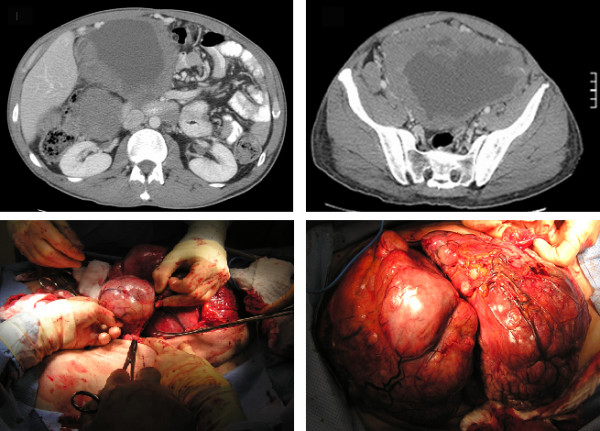
**1a and b- **CT scan of the abdomen and pelvis with oral and intravenous contrast demonstrating a large, multicentric GIST with extensive collateral circulation extending from the upper abdomen to the pelvis. **1c and d- **Intraoperative photos of the mass being delivered from the abdomen.

An abdominal computed tomography demonstrated a large, multicentric tumor with extensive collateral circulation (Fig 1a and 1b). Preoperative selective embolization of associated vessels was performed to reduce operative bleeding. The following day, extensive tumor debulking of both the dominant mass and 99% of the remaining multifocal 40 cm × 25 cm × 10 cm GIST was achieved (Fig 1c and 1d), obtaining an R1 resection. Immediately after completing the resection, the patient became euglycemic without requiring intravenous glucose. The patient was euglycemic, active and without radiographic evidence of progression 6 months after his surgery. Due to gastrointestinal intolerance to imatinib, he is currently on therapy with sunitinib with stable disease.

**Figure 2:**
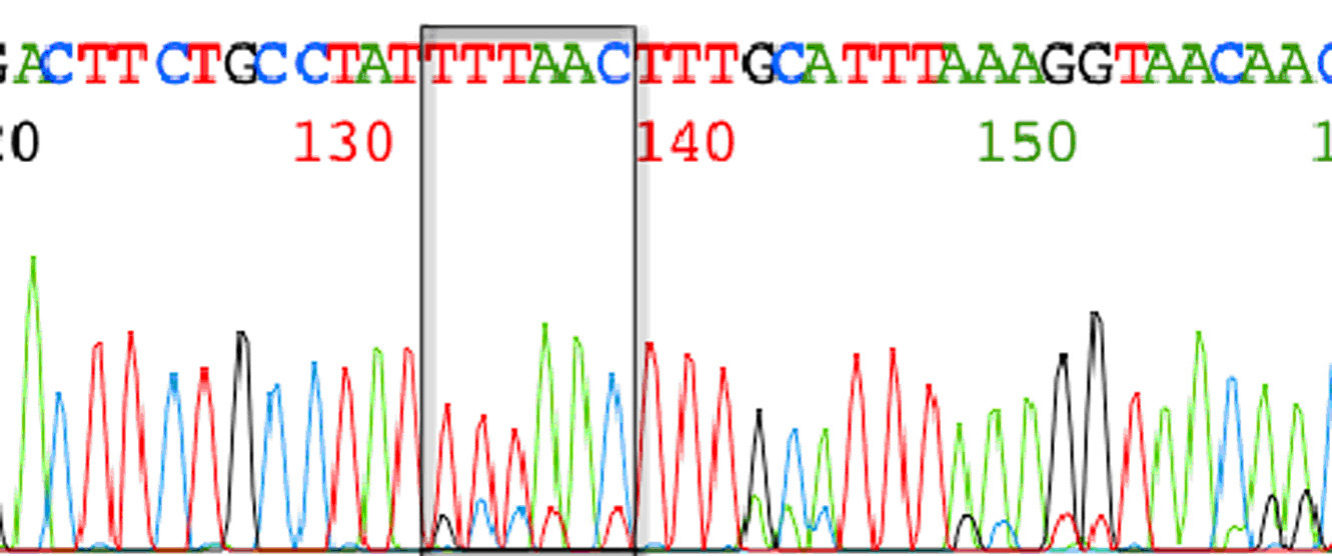
Electrophergram of the mid portion of exon 9 of KIT from this patient's GIST showing two alleles, one with a 6 bp tandem repeat insertion (GCCTAT) leading to a duplication of amino acids alanine and tyrosine at codons 502 and 503 (enclosed in the square).

Pathologic analysis identified the presence of KIT (CD-117) in the tumor; confirming GIST histology. Extracted DNA was amplified by polymerase chain reaction for exons 9, 11, 13 and 17 of KIT. No mutations were found in exons 11, 13 or 17; however one allele at position 1530 in exon 9 contained a 6 bp tandem repeat insert (GCCTAT). This led to a duplication of amino acid residues alanine 502 and tyrosine 503 (Fig 2). The mutation was confirmed by a TOPO cloning system (Invitrogen, Carlsbad, California USA), which detected the presence of one normal allele and one with the 6 bp insert as described.

**Figure 3:**
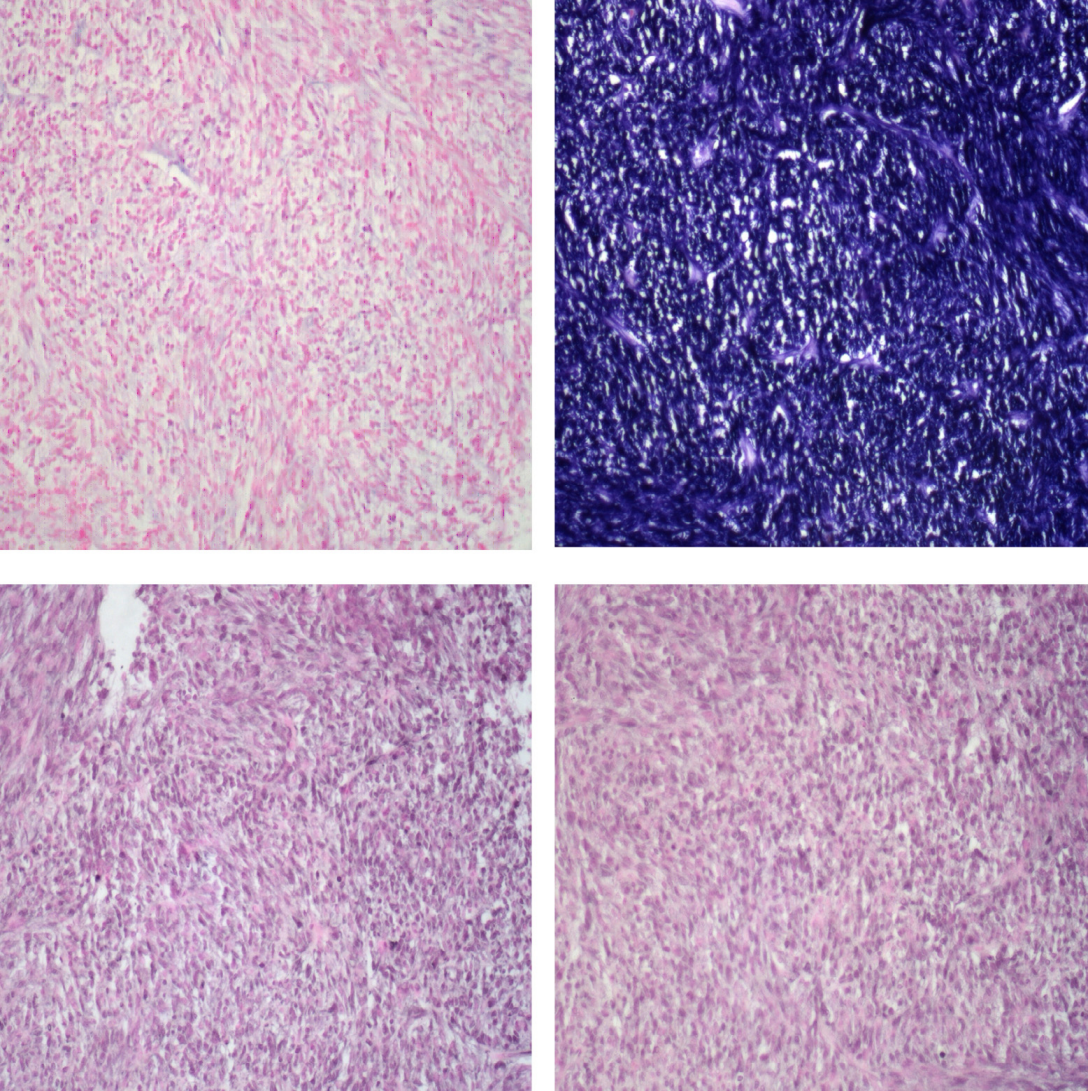
**3a- **In situ hybridization: IGF-II mRNA sense probe (200×). **3b- **In situ hybridization: IGF-II mRNAantisense probe (200×). **3c- **Immunohistochemistry: Polyclonal rabbit anti E [68–88] domain of human pro-IGF-II ("big" IGF-II) (dilution 1:50) (200×). **3d- **Immunohistochemistry: Polyclonal rabbit anti-human IGF-II (dilution 1:100) (200×).

The patient's serum and tumor were sent to the University Medical Center in Utrecht (The Netherlands) for further analysis. The concentration of II E [68–88] ("big" IGF-II) in plasma was 51.9 ng/mL (normal: 6.5–17.5 ng/mL). The levels of IGF binding protein 6 (IGFBP-6) were reported to be within the normal range. In situ hybridization of the tumor samples demonstrated high expression of IGF II mRNA and elevated pro-IGF II (figure 3).

It has been found that specific KIT mutations in GIST may predict a patient's prognosis. Mutations on exon 11 are the most common (79%), while those on exon 9 are only found in about 11% of GIST. Most patients with exon 11 mutations respond to treatment with imatinib, while less than half of those with exon 9 mutations respond and are almost twice as likely to recur [16-18].

The particular mutation we found in our patient has been reported very infrequently, and is thought to induce a ligand-independent gain-of-function with homodimerization and constitutive autophosphorylation [19,20]. The significance of the association of this mutation and non-islet cell tumor hypoglycemia (NICTH) has yet to be established.

The incidence of NICTH is thought to be one fourth that of functional insulinomas (1 case of NICTH per million people-years) [21,22]. NICTH usually occurs with large or metastatic tumors that are usually of mesenchymal origin, but may also present in other types of cancer [23]. Searching via MEDLINE, PubMed, Gold Rush and article cross-referencing, we only found 4 other publications of NICTH in GIST; four of which had overexpression of "big" IGF II and in two there was overexpression of pro-IGF II [9-12].

High concentrations of pre-pro-IGF II genes that are not properly glycosylated result in the high molecular weight "big" IGF II. Compared to physiologic IGF II, it has a significantly higher affinity to the insulin receptor, and a lower affinity to its binding protein [24,25]. The association of "big" IGF II and NICTH was first identified by Daughaday in a patient with a fibrosarcoma [26]. Non-islet cell tumor-induced hypoglycemia secondary to "big" IGF II is difficult to confirm because laboratory tests for it are not readily available. A landmark observation by Teale et al. was that IGF I and II levels in patients with hyperinsulinemic hypoglycemia were both usually low, while patients with NICTH had normal levels of IGF II [25]. Consequently, Hizuka et al. demonstrated that IGF I was always low in hypoglycemic patients with "big" IGF II, while those without elevated "big" IGF II had both low IGF I and II [27].

The management of most patients with NICTH requires prompt intravenous hydration and glucose infusions. Once stable, the ideal treatment is to remove the source of hypoglycemia. In NICTH, the hypoglycemia is not due to a dysfunctional insulin receptor nor abnormal K-ATP channels, therefore diazoxide is unlikely to be effective [28]. Attempts with imatinib as a first-line therapy may fail due to resistance to the drug, or take as long as 5 weeks before being able to interrupt the intravenous glucose infusions [10]. A recent report by Hamberg et al. [12] found in one patient with NICTH from GIST that elevated concentrations of imatinib seemed to worsen hypoglycemia, while another patient spontaneously resolved without any GIST-related therapy. Alternative nonoperative cytoreductive techniques such as selective embolization and radiofrequency ablation may have a role in alleviating hypoglycemia; however these have not been reported in the literature. Additionally, they may potentially result in greater circulating "big" IGF II during necrosis of the GIST and may explain Hamberg's findings in a patient treated with imatinib alone that had hypoglycemia proportional to the dose of imatinib. In other (non GIST) cases of NICTH, low dose steroids and growth hormone have allowed palliative weaning from intravenous glucose [25,29,30]. Surgical resection remains as the most rapid and cost-effective therapy to normalize glucose metabolism in most cases of NICTH.

## Conclusion

Our experience and review of the literature suggest that a multidisciplinary approach is helpful in the management of NICTH in GIST and that prompt surgical debulking is the treatment of choice.

## Competing interests

The author(s) declare that they have no competing interests.

## Authors' contributions

GAE conceived and drafted the manuscript, obtained references, participated in its design and coordination and edited the figures and legends. WAR directed and obtained the GIST mutational analysis and participated in its design and editing. TLN participated in its drafting, references, editing and design. DCH contributed to the patient's care and contributed to the conception and drafting of the manuscript. GJW Performed the GIST mutational analysis and participated in its design and coordination. LB contributed to the patient's care and coordinated communication with the University Medical Center Utrecht, Utrecht, The Netherlands for obtaining molecular analysis of IGF. RG contributed to the patient's care and participated in its editing, design and coordination. MDM contributed to the patient's care, performed surgical resection, obtained operative photos and participated in its design, editing and coordination. All authors read and approved the final manuscript.

## Pre-publication history

The pre-publication history for this paper can be accessed here:



## Figures and Tables

**Table 1:** Initial Evaluation for Sources of Hypoglycemia

	**Patient's Serum**	**Normal Range**
**Insulin**	2 μU/mL	0–20 μU/mL
**Pro Insulin**	2.4 pmol/L	2.1–26.8 pmol/L
**C-peptide**	0.6 ng/mL	1.1–4.6 ng/mL
**Cortisol**	18 μg/dL	5–25 μg/dL
**Chlopropamide**	0 μg/mL	0 μg/mL
**Tolazamide**	0 μg/mL	0 μg/mL
**Tolbutamine**	0 μg/mL	0 μg/mL
**Gyburide**	0 μg/mL	0 μg/mL
**Glipizide**	0 μg/mL	0 μg/mL
**Acetohexamide**	0 μg/mL	0 μg/mL
**IGF Binding Protein-3**	1180 ng/mL	1237–2604 ng/mL
**IGF-I**	40 ng/mL	90–360 ng/mL
**IGF-II**	391 ng/mL	288–736 ng/mL

IGF = Insulin-like growth factor
